# Clinical Characteristics of Patients With AIDS and *Talaromyces marneffei* Infection of the Central Nervous System: A Retrospective Observation Study

**DOI:** 10.1155/arat/9954449

**Published:** 2026-07-31

**Authors:** Yunmei Li, Jinhong He, Xiangxi He, Yinshuang Peng, Xingxing Luo, Xiaoxin Xie, Yanhua Fu, Hai Long

**Affiliations:** ^1^ Infection Department, Guiyang Public Health Clinical Center, Guiyang 550004, China

**Keywords:** AIDS, HIV, intracranial infection, metagenomic next-generation sequencing, *Talaromyces marneffei*

## Abstract

**Objective:**

To analyze the clinical characteristics of patients with acute immunodeficiency syndrome (AIDS) combined with *Talaromyces marneffei* (TM) infection of the central nervous system (CNS), thereby improving awareness toward early diagnosis and treatment.

**Methods:**

The clinical data of eight patients with AIDS who were treated for CNS TM infection in the Guiyang Public Health Treatment Center from May 2021 to November 2022 were retrospectively analyzed.

**Results:**

The median age of the patients was 43.50 (range: 35.00–58.00) years, and all eight were male. TM infection was confirmed via metagenomic next‐generation sequencing (mNGS) in three cases, positive cerebrospinal fluid (CSF) cultures of TM in four cases, and both in one case. CSF and blood cultures were both positive for one patient, whereas multiple blood cultures were negative for the other seven. The number of nucleated cells and the protein level in the CSF were elevated in five and six patients, respectively, and the CSF levels of glucose and chloride were low in four patients each. Seven patients had intracranial lesions upon head imaging, and all eight were discharged from the hospital with improvement after antifungal treatment. The median CD4+ T‐cell count was 58.50/μL (range: 39.00–73.00/μL), indicating severe immunosuppression.

**Conclusion:**

The clinical characteristics and CSF‐related examinations of patients with AIDS combined with CNS TM infection are not distinct, complicating diagnosis and increasing the likelihood of misdiagnosis. Early diagnosis and systemic antifungal therapy can improve patients’ prognosis.

## 1. Introduction

*Talaromyces marneffei* (TM) infection is an opportunistic infection and the third most common infection among patients with acquired immunodeficiency syndrome (AIDS), after tuberculosis and cryptococcosis/pneumocystis pneumonia [1]. Poor compliance with HAART is associated with a heavy disease burden and high mortality [2, 3].

TM infections can be divided into localized and disseminated types according to the site and characteristics of the disease. In localized infections, the pathogen is limited to the invasion site and only affects individual organs, whereas in the disseminated type, the pathogen invades multiple organs. TM infections in patients with AIDS are mostly of the disseminated type [4]. Concurrent HIV infection and central nervous system (CNS) involvement by TM is rare, and its diagnosis is challenging. Historically, the prognosis for such patients has been poor due to diagnostic difficulties and delayed treatment initiation [5]. However, emerging evidence suggests that with early diagnosis and systemic antifungal therapy, the outcomes for these patients can be substantially improved [6, 7]. Therefore, early diagnosis and prompt treatment of CNS TM infection in AIDS patients remain critical clinical priorities. This article reviews the clinical characteristics of eight patients with AIDS and CNS TM infection who were diagnosed early and treated systematically, aiming to provide a reference for clinical practice and improve patient prognosis. This report provides a reference for the clinical diagnosis and treatment of such patients to improve their prognosis.

## 2. Patients and Methods

### 2.1. Patients

We retrospectively analyzed the medical records of eight patients with AIDS who were admitted to Guiyang Public Health Treatment Center for CNS TM infection between May 2021 and November 2022.

The diagnosis of AIDS with CNS TM infection was established based on the following criteria: (1) AIDS was diagnosed according to the Chinese Guidelines for Diagnosis and Treatment of HIV/AIDS (2021 edition) [5]; (2) CNS involvement was clinically suspected based on symptoms, signs, or imaging findings; (3) TM infection was confirmed by at least one of the following microbiological methods: metagenomic next‐generation sequencing (mNGS) of cerebrospinal fluid (CSF), positive culture of TM from CSF, peripheral blood, or bone marrow; and (4) neuroimaging revealed intracranial lesions consistent with infection.

### 2.2. Methods

Clinical data were retrospectively collected. Two doctors consulted the patients’ medical records from the hospital information system and collected relevant data, including patients’ general condition, clinical symptoms, laboratory test results, treatment plan, disease outcome, follow‐up, and survival. We declare that we did not use any artificial intelligence–generated content (AIGC) tools in the manuscript.

## 3. Results

### 3.1. General Information and Clinical Manifestations

The median age of the patients was 43.50 (range: 35.00–58.00) years. All eight patients were male, six were unemployed, one was a worker, and one was a staff member. Five patients had symptoms of a CNS infection, including headache, mental and behavioral abnormalities, and uni‐ or bilateral lower‐limb movement disorders; the other three had no neurological symptoms and presented with other symptoms (fever, fatigue, abdominal pain, and skin ulcers). Meningeal stimulation was positive in two cases, as shown in Table 1.

**TABLE 1:** Clinical features of TM CNS infection in the eight patients.

	Age (y)	Chief complaint	Neurological symptoms	Meningeal irritation	Other manifestations
1	58	Headache, fever for 1 month	Headache	Negative	Fatigue, poor appetite
2	36	Fever for 1 day	None	Negative	None
3	35	Headache for 1 month	None	Negative	Abdominal pain
4	50	Left‐limb weakness for 5 days	Unsteady gait, weak grip, numbness of the right limbs	Positive	None
5	47	Poor and weak for 2 months	None	Negative	Lethargy
6	40	Numbness and weakness of the limbs and trunk for 4 months	None	Positive	Poor appetite
7	38	Acute mental status change for 1 day	Psychobehavioral abnormalities	Negative	Fever
8	49	Penile ulcer for 1 month	None	Negative	None

### 3.2. CSF and Laboratory Examinations

The CSF was clear in seven patients and yellow in one. The intracranial pressure was elevated in two patients (> 180 mmH_2_O) and normal in six. Elevated levels were observed in the number of nucleated cells in the CSF in five cases, CSF protein in six cases, and CSF microprotein in seven cases. Low levels of CSF glucose and CSF chloride were detected in four cases each. TM was detected in three cases via CSF mNGS, four cases via CSF culturing, and one case via both CSF culturing and mNGS. Both CSF and blood cultures were positive in one of the eight patients, and multiple blood cultures were negative in the other seven. Smears were negative for CSF bacteria, *Cryptococcus neoformans*, and mycobacteria in all cases, as was TB culturing. The median CD4+ T‐cell count was 58.50 (range: 39.00–73.00)/μL, and the median CD4+/CD8+ ratio was 0.14 (range: 0.06–0.51), as shown in Table 2.

**TABLE 2:** Laboratory results of TM central nervous system infection in eight patients.

Index	1	2	3	4	5	6	7	8
Intracranial pressure (mmH_2_O)	150.00	220.00	70.00	260.00	120.00	90.00	150.00	100.00
CSF color	Limpid	Limpid	Limpid	Limpid	Limpid	Pale yellow	Limpid	Limpid
Nucleated cells in CSF (≤ 8 × 10^6^)	12	6	30	58	2	48	74	4
CSF protein	Positive	Positive	Positive	Positive	Positive	Positive	Positive	Positive
CSF glucose (2.50–4.50 mM)	1.89	2.77	0.84	4.43	2.91	< 0.30	1.20	3.68
CSF chlorine (120–130 mM)	110.00	128.00	115.00	125.00	117.00	80.00	121.00	127.00
CSF microprotein (8.00–43.00 mg/dL)	181.50	44.40	178.40	153.60	142.00	14.20	166.00	46.00
CD4+ T‐lymphocytes (/μL)	69	73	65	40	41	52	39	66
Blood culture	Negative	Negative	Negative	Negative	TM	Negative	Negative	Negative
CSF culture	Negative	TM	TM	TM	TM	Negative	TM	Negative
CSF mNGS	TM	—	—	—	—	TM	TM	TM
Viral load (copies/mL)	283,000	494,000	158,000	613,000	123,000	265,000	249,000	48,764

### 3.3. Imaging Analysis

**FIGURE 1:**
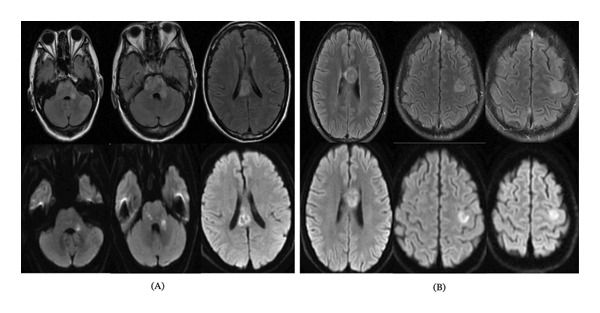
Enhanced MRI of the head of Patient 1 (A) and Patient 3 (B).

Seven of the eight patients presented with various intracranial abnormalities on neuroimaging, manifesting as circular or nodular enhancing lesions or mild uniform enhancement (see examples in Figure 1). In two patients, the lesions were accompanied by perifocal edema. The remaining patient showed no detectable intracranial abnormalities. Thoracic CT revealed pulmonary abnormalities in all eight patients, indicating disseminated TM infection in this cohort.

### 3.4. Treatment Outcome

All 8 patients received antifungal therapy. Six patients were tentatively diagnosed with tuberculous meningitis. For these six patients, antituberculosis therapy was administered in the early stage of the disease according to their clinical manifestations and results of routine CSF and biochemical tests, among other methods of detection, but their condition did not improve. After detection of TM upon CSF culturing or mNGS, their treatment was adjusted and their condition improved. Five patients received amphotericin B induction therapy for more than 4 weeks, until their CSF fungal cultures were negative, and the other three received voriconazole induction therapy. All eight patients were given oral itraconazole consolidation therapy (400 mg/day) and maintenance therapy (200 mg/day) until secondary prophylaxis was discontinued after their CD4 count was greater than 100/μL for 6 months. All eight patients were treated with combination antiretroviral therapy (cART) at the 4th week of treatment. The median hospital stay of the eight patients was 45 (20–64) days, and all of them were alive 3 years after discharge.

## 4. Discussion

TM infection of the CNS is rarely reported and is only described in studies with small samples or case reports. Systemic talaromycosis in the context of HIV lacks characteristic signs and symptoms making it difficult to differentiate from other infectious diseases. This diagnostic ambiguity places a heavy reliance on clinical suspicion, particularly in severely immunocompromised patients residing in or originating from endemic regions. Le et al. [8] analyzed 21 cases of TM infection of the CNS. Only five patients had neurological symptoms upon admission, whereas the other 3 had fever, poor intake, and penile ulcer. In this study, five of the eight patients with TM infection of the CNS had CNS symptoms; two had fatigue, poor appetite, fever, and abdominal pain; and one had a penile skin ulcer, generally corresponding with the observations of Le et al. [8]. These findings underscore a critical diagnostic challenge: The absence of neurological symptoms does not rule out CNS involvement. Clinicians must maintain a high index of suspicion for CNS TM infection even in AIDS patients presenting with seemingly mundane symptoms, particularly when accompanied by profound immunosuppression.

The CD4+ T‐lymphocyte count is very low in patients with CNS TM infection. Most of the 38 patients analyzed by Le et al. [8] had CD4+ T‐lymphocyte counts lower than 50/μL, with a mean count of 11 (standard deviation, 10.12)/μL. The median CD4+ T‐cell count of the eight patients in this study was 58.50 (range: 39.00–73.00)/μL, which was consistent with their report. Le et al. [8] concluded that the intracranial pressure of patients with CNS TM infection was mild to moderate; the mean intracranial pressure of the 38 patients was 212.5 (range: 150.0–322.5) mmH_2_O. The mean intracranial pressure in this study was higher, at 135 (range: 70–260) mmH_2_O.

In this analysis, five patients had a high CSF protein concentration and low CSF glucose and chloride concentrations, which was consistent with the results of a previous study [9]. This CSF profile—lymphocytic pleocytosis with elevated protein and low glucose—closely resembles that of tuberculous meningitis and cryptococcal meningitis, further complicating the differential diagnosis. In clinical practice, cryptococcal meningitis can be readily identified through India ink staining, cryptococcal antigen testing, and fungal culture [10]. However, the detection rate of *Mycobacterium tuberculosis* via CSF acid‐fast staining, PCR, or culturing is very low [11], and CNS TM infection may be misdiagnosed as tuberculous meningitis when the pathogen cannot be identified. In this study, six patients were tentatively diagnosed with tuberculous meningitis in the early stage of the disease according to their clinical manifestations and routine CSF, biochemical, and other test results. Antituberculous treatment did not improve their condition, but TM was detected via CSF culturing or CSF mNGS, and treatment for TM did improve their condition. This clinical scenario—suspected tuberculous meningitis failing to respond to antituberculous therapy—should prompt immediate consideration of alternative etiologies, including CNS TM infection.

This study provides the first detailed description of neuroimaging features in a series of patients with CNS TM infection. Seven of eight patients demonstrated intracranial lesions on imaging, characterized by circular or nodular enhancement or mild uniform enhancement. Two patients exhibited perifocal edema. Previous literature has been limited to isolated case reports describing ventricular dilatation [8], cerebrovascular inflammation, and hemorrhagic cerebral infarction [12]. Our findings suggest that these enhancement patterns, while not pathognomonic, may represent characteristic imaging features of CNS TM infection. However, given the small sample size, larger studies are needed to validate these observations and define the full spectrum of neuroimaging abnormalities in this disease.

The nonspecific clinical, laboratory, and imaging features of CNS TM infection delay diagnosis. The current diagnostic gold standard remains CSF culture or histopathological identification of TM in blood or brain tissue [13]. CSF culture has demonstrated high sensitivity in some series; Le et al. [8] reported positive CSF cultures in 89.5% (34/38) of patients. In our study, CSF culture was positive in 62.5% (5/8) of patients. Notably, in the three culture‐negative patients, TM was detected by CSF mNGS. This highlights the critical role of mNGS as a supplementary diagnostic tool, offering superior sensitivity and faster turnaround time compared to traditional culture [1], particularly in cases where clinical suspicion is high but conventional testing is unrevealing.

According to the expert consensus on the diagnosis and treatment of HIV/AIDS complicated with talaromycosis (2024 updated version), when TM involves the CNS, 5 mg/kg liposomal amphotericin B should be administered, once a day, for an induction period of 4–6 weeks. Voriconazole is an effective and relatively well‐tolerated alternative treatment regimen. Moreover, in this study, oral itraconazole was administered (200 mg) twice a day for 10 weeks during the consolidation period, and once a day during the maintenance period, until cART could be administered and the CD4+ T‐cell count was greater than 100/μL for 6 months, and the drug could be safely stopped. Once the CD4+ T‐cell count drops below 100/μL again, preventive therapy should be restarted. In this study, five of the eight patients were treated with amphotericin B induction, and the other three were admitted owing to symptoms of bone‐marrow suppression and treated with voriconazole induction [14]. All eight were treated with itraconazole consolidation and preventive treatment, and all were discharged with improvement. Mei‐Qin et al.[15] revealed that, if timely antifungal treatment is not available for patients with AIDS combined with CNS TM infection, the mortality rate is high. In the study by Le et al.[8], 36.8% (14/38) of patients did not receive timely antifungal treatment and died. Li et al.[9] reported that all nine patients treated with amphotericin B followed by itraconazole in the treatment of TM infection of the CNS improved and were discharged from hospital. In this study, all eight patients were definitively diagnosed in the early stage of the disease, treated with systemic and standardized antifungal therapy, and started on cART during the 4th week of antifungal therapy. They all improved, were discharged, and were still being followed up 3 years later. These contrasting outcomes underscore a pivotal message: CNS TM infection is not uniformly fatal. With early recognition and appropriate therapy, excellent outcomes are achievable. The critical barrier remains timely diagnosis—a goal that requires sustained clinical vigilance.

In summary, the clinical manifestations, CSF findings, and imaging features of AIDS patients with CNS TM infection are nonspecific, rendering early diagnosis difficult and misdiagnosis common. Familiarity with this disease entity—particularly the recognition that CNS involvement can occur in the absence of neurological symptoms and that imaging may reveal characteristic enhancement patterns—is essential for timely diagnosis. Until more specific diagnostic biomarkers emerge, a high index of suspicion remains the most powerful tool clinicians possess to ensure that patients receive life‐saving treatment.

## 5. Conclusion

The clinical characteristics and CSF‐related examinations of patients with AIDS combined with CNS TM infection are not distinct, complicating diagnosis and increasing the likelihood of misdiagnosis. Early diagnosis and systemic antifungal therapy can improve patients’ prognosis.

## Author Contributions

Conceptualization: Yunmei Li and Hai Long.

Resources: Yunmei Li, Jinhong He, and Xiangxi He.

Writing–original draft: Yunmei Li.

Writing–review and editing: Hai Long.

Supervision: Hai Long and Xiaoxin Xie.

Validation: Yanhua Fu.

Methodology: Xingxing Luo and Yinshuang Peng.

Data curation: Xiaoxin Xie.

## Funding

This work was supported by the Enhancement of Medical Services and Guarantee Capacity (Capacity Building of Medical and Health Institutions) Central Subsidy Fund (National Clinical Key Specialty Capacity Building Project: Guiyang Public Health Clinical Center) (Project Code: 10000015Z155080000004; Document Number: Qian Cai She [2025] No. 174).

## Ethics Statement

The study was performed according to the principles of the Declaration of Helsinki, and prior approval was obtained from the Institutional Review Board of Research Center.

## Conflicts of Interest

The authors declare no conflicts of interest.

## Data Availability

The datasets used or analyzed during the current study are available from the corresponding author on reasonable request.

## References

[1] Limper A. H. , Adenis A. , Le T. , and Harrison T. S. , Fungal Infections in HIV/AIDS, Lancet Infectious Diseases. (2017) 17, no. 11, e334–e343, 10.1016/s1473-3099(17)30303-1. 28774701

[2] Le T. , Wolbers M. , Chi N. H. et al., Epidemiology, Seasonality, and Predictors of Outcome of AIDS-Associated Penicillium Marneffei Infection in Ho Chi Minh City, Viet Nam, Clinical Infectious Diseases. (2011) 52, no. 7, 945–952, 10.1093/cid/cir028. 21427403 PMC3106230

[3] Larsson M. , Nguyen L. H. , Wertheim H. F. et al., Clinical Characteristics and Outcome of Penicillium Marneffei Infection Among HIV-Infected Patients in Northern Vietnam, AIDS Research and Therapy. (2012) 9, no. 1, 24–28, 10.1186/1742-6405-9-24. 22897817 PMC3439243

[4] 13th Five-Year National Science and Technology Major Special AIDS Opportunistic Infection Research Group. Expert Consensus on Clinical Diagnosis and Treatment of AIDS Complicated With Marneffei Basiform Mycosis, Journal of Southwest University. (2019) 42, no. 7, 61–75.

[5] Zhou Y. , Lu Y. , and Chen Y. , Diagnosis and Treatment Status and Research Progress of AIDS Complicated With Cyanobacteria Marneffei, Chinese Journal of Mycoliology. (2019) 14, no. 5, 308–312.

[6] Hiv/Aids Hepatitis C Group , Infectious Diseases Branch, Chinese Medical Association, Chinese Center for Disease Control and Prevention. China AIDS Diagnosis and Treatment Guidelines (2021 Edition), China AIDS STD. (2021) 27, no. 11, 1182–1201.

[7] Chakrabarti A. and Slavin M. A. , Endemic Fungal Infections in the Asia—Pacific Region LJJ, Medical Mycology. (2011) 49, no. 4, 337–344, 10.3109/13693786.2010.551426. 21254966

[8] Le T. , Huu Chi N. , Kim Cuc N. T. et al., AIDS-Associated Penicillium Marneffei Infection of the Central Nervous System, Clinical Infectious Diseases. (December 15 2010) 51, no. 12, 1458–1462, 10.1086/657400. 21054180 PMC3106247

[9] Li Y. Y. , Dong R. J. , Shrestha S. et al., AIDS-Associated *Talaromyces marneffei* Central Nervous System Infection in Patients of Southwestern China, AIDS Research and Therapy. (May 26 2020) 17, no. 1, 10.1186/s12981-020-00281-4. PMC7249401 32456686

[10] Zhu W. , Aixin Li , Huang X.e et al., HIV Merger of Cryptococcal Meningitis Research Progress, China AIDS Sexually Transmitted Diseases. (2021) 27, no. 5, 561–5633, 10.13419/j.carolcarrollnkiAIDS.2021.05.32.

[11] Zhang G. L. , Su H. Y. , Yang L. et al., Clinical Analysis of 36 Cases of AIDS Complicated With Disseminated Penicilliosis Marneffei, Infectious Diseases Information. (2015) 28, no. 06, 369–371.

[12] Gao Y. , Qu M. , Song C. , Yin L. , and Zhang M. , Cerebral Vasculitis Caused by *Talaromyces marneffei* and Aspergillus Niger in a HIV-Positive Patient: A Case Report and Literature Review, Journal of NeuroVirology. (April 2022) 28, no. 2, 274–280, 10.1007/s13365-021-01032-5. 34981436 PMC9187570

[13] Valero C. , Martín-Gómez M. T. , and Buitrago M. J. , Molecular Diagnosis of Endemic Mycoses, Journal of Fungi. (2022) 9, no. 1, 10.3390/jof9010059. PMC9866865 36675880

[14] Expert Consensus on the Diagnosis and Treatment of HIV/AIDS With Marneffei Basiform Mycosis (2024 Update), China AIDS sexually transmitted diseases. (2024) 30, no. 6, The, 563–572, 10.13419/j.carolcarrollnkiAIDS.2024.06.02.

[15] Mei-Qin C. , Wei-Li Lu , Shun W. et al., A Case of Central Nervous System Infection of AIDS With Marneffei Bacillus, Chinese Journal of Infectious Diseases. (2021) 39, no. 11, 3–7.

